# Jitter noise modeling and its removal using recursive least squares in shape from focus systems

**DOI:** 10.1038/s41598-022-18150-7

**Published:** 2022-08-18

**Authors:** Husna Mutahira, Vladimir Shin, Unsang Park, Mannan Saeed Muhammad

**Affiliations:** 1 Department of Computer Science and Engineering, Sogang University, Seoul, 04107 South Korea; 2 Department of Information and Statistics, Research Institute of Natural Science, Gyeongsang National University, Jinju, 52828 South Korea; 3 Department of Electrical and Computer Engineering, Sungkyunkwan University, Suwon, 16419 South Korea

**Keywords:** Engineering, Mathematics and computing, Optics and photonics, Physics

## Abstract

Three-dimensional shape recovery from the set of 2D images has many applications in computer vision and related fields. Passive techniques of 3D shape recovery utilize a single view point and one of these techniques is Shape from Focus or SFF. In SFF systems, a stack of images is taken with a single camera by manipulating its focus settings. During the image acquisition, the inter-frame distance or the sampling step size is predetermined and assumed constant. However, in a practical situation, this step size cannot remain constant due to mechanical vibrations of the translational stage, causing jitter. This jitter produces Jitter noise in the resulting focus curves. Jitter noise is invisible in every image, because all images in the stack are exposed to the same error in focus; thus, limiting the use of traditional noise removal techniques. This manuscript formulates a model of Jitter noise based on Quadratic function and the Taylor series. The proposed method, then, solves the jittering problem for SFF systems through recursive least squares (RLS) filtering. Different noise levels were considered during experiments performed on both real as well as simulated objects. A new metric measure is also proposed, referred to as depth distortion (DD), which calculates the number of pixels contributing to the RMSE in percentage. The proposed measure is used along with the RMSE and correlation, to compute and test the reconstructed shape quality. The results confirm the effectiveness of the proposed scheme.

## Introduction

In recent years, extensive research has been conducted towards the recovery of 3D information from its corresponding 2D images. Given a set of images, important depth information can be effectively obtained and further used for 3D reconstruction. This information is used in various applications, such as robotic manipulation, automatic inspection, medical imaging, microscopy, consumer cameras, bioinformatics etc.^1–6^

**Figure 1:**
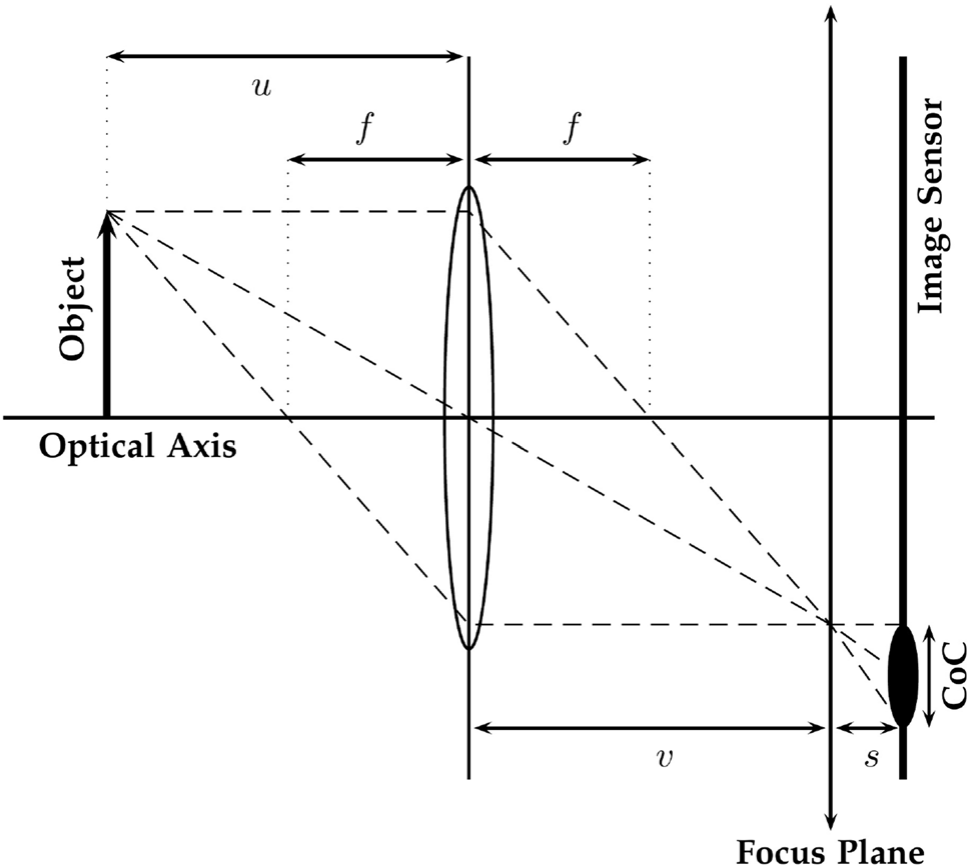
Focusing via Gaussian lens law.

Shape from Focus (SFF) is the process of reconstructing the depth of the scene by actively changing the camera optics until the point of interest is in focus. It is one of the passive techniques that uses one camera for 3D shape reconstruction. The SFF system must record a large sequence of image frames of the object/scene that correspond to different levels of the object/scene in focus^7^. The camera optics can be changed by changing the lens position or the object position relative to the camera. The depth of the focused point can be obtained through the thin lens Gaussian law:

1 $$\begin{aligned} \frac{1}{f}=\frac{1}{u}+\frac{1}{v}, \end{aligned}$$

wherein *f* denotes the focal length of the imaging device. The distance of the object point from the imaging device is given by *u*, and the position of the object point where it is best focused by the lens is represented by *v*, and is given in Fig. 1.

**Figure 2:**
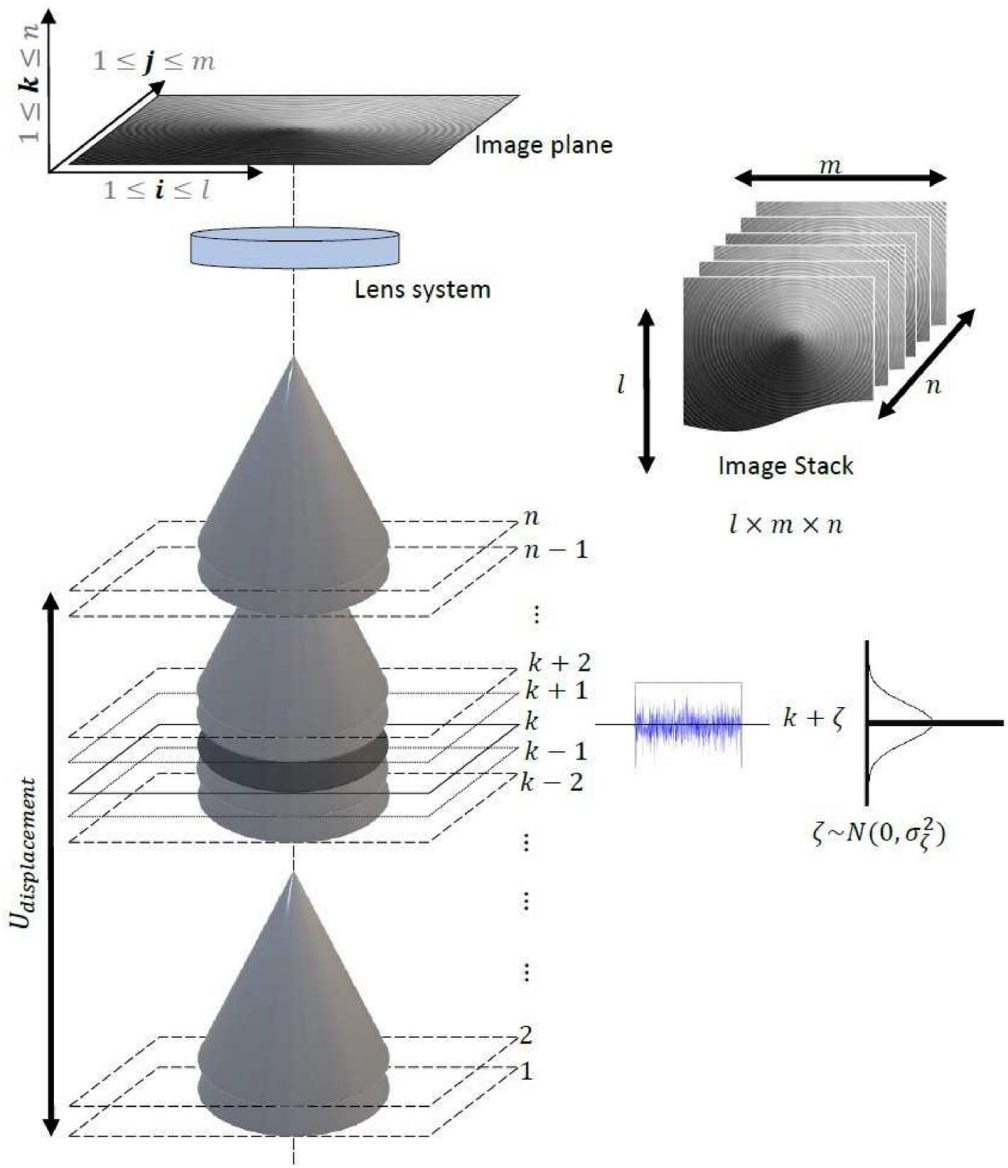
Image acquisition in SFF systems.

Images can be obtained by manipulating any of the above-mentioned factors, but typically *u* of the system is varied to acquire images. The optical microscope is an example of such type of a system. Nonetheless, for the SFF systems the magnification of the imaging system should be constant and the depth of field should be as shallow as possible^8^. After image acquisition, the resulting image stack *I* is represented by $l\times m\times n$ dimensions. Each pixel in the stack is represented by $P_{(i,j)}(k)$, where $1\le i\le l$, $1\le j\le m$ and $1\le k\le n$ are the indices in the *l*, *m*, and *n* directions. $P_{(i,j)}(k)$ also represents the pixel curve along the optical axis. This is shown as in Fig. 2. The number of images (*n*) can be calculated by:

2 $$\begin{aligned} n = \frac{U_{disp}}{\Delta }, \end{aligned}$$

where $\Delta$ in the above equation represents the sampling step size and $U_{disp}$ represents the total displacement of the translational stage during image acquisition^9^. For changes in *u*, the step size expression is provided by Muhammad and Choi^9^.

Since SFF algorithms use image focus analysis to recover 3D shape of an object, a focus measure (FM) is calculated over small image regions of the image frame in the image sequence. The value of the FM increases with the increase in image sharpness and achieves a maximum value when the image is best focused^10^. Since the advent of SFF methods, various FMs have been proposed to measure the degree of focus present in the image. Their performance can be inflcuenced under various conditions, such as image noise level, contrast, saturation and window size, and the effects of these conditions on FMs have been extensively studied in recent years^11–13^.

A brief introduction of popular FMs used for comparison in this manuscript is given here: Sum of Modified Laplacian (*SML*), and Tenengrad or Tenenbaum (*TENG*) use squares of the second derivative of the image and first derivative of the image, respectively^13,14^. Gray Level Variance (*GLVA*) is a statistical method to compute variance as focus measurement^11^ whereas Image Curvature (*CURV*) calculates the image surface curvature. Some other FMs^13^ used for experiments in this study include Image Contrast (*CONT*), 3D Laplacian (*GRA-3*), Discrete Cosine Transform Energy (*DCTE*), Spatial Frequency (*SFRQ*), Sum of Wavelet Coefficients (*WAVS*), and Steerable Filters (*SFIL*).

After FM application an initial depth map estimate is obtained which can be further improved by refinement procedures. Traditional SFF techniques utilize SML and apply Gaussian interpolation to compute intra-frame values for better focus^15,16^. Piecewise curved surface approximation^17,18^ was used for the focused image surface and curved focused image surface, respectively. Traditional and Deep Neural Networks^1,19,20^ were introduced by various authors to enhance the results of SFF techniques. Another method to improve the efficiency of neural networks, by introducing the weight passing method, was also employed^21^. Pulsed coupled neural network to aggregate shape using focus was given by Yan et al.^22^. A method based on Bezier surface approximation^8^ was proposed to approximate the resultant shape through Bezier polynomials. Another method of applying 3D weighted least squares^23^ to enhance image focus volume was presented so to increase the accuracy of 3D shapes. The wavelet transform method^24^ was also used to improve shape reconstruction. Guided image filtering^25^ is also employed for depth enhancement in SFF.

Recovery of several 3D shapes by applying different FMs^26^, and combining their results into one final shape is also one of the popular techniques. A combination of 3D steerable filters^27^ on treating texture-less regions were also utilized to enhance the texture-less surface reconstruction. Another SFF method based on the analysis of 3D structure tensor of the image sequence was proposed by Mahmood et al.^28^. A method for depth reconstruction that used non-local matting Laplacian along with Markov random field was developed by Ma et al.^29^. Shape optimization through non-parametric regression is presented by Jang et al.^30^. Adaptive sum of weighted modified Laplacian is also proposed^31^.

## Focus measurement and focus curve modeling

Focus measurement is the main concept in the SFF systems, and the sharpness criterion used to evaluate the level of focus in an image is called the focus measure operator (FM)^15^. The principle behind FM is to respond to the high-frequency content in the image, and ideally it should produce maximum response when the image is focused^32^. The main objective of FM in SFF systems is to provide a sharp focus curve (in parallel to the optical axis) for every object point in the image stack^33^. The FM is applied to each pixel of the image stack, as given in the following equation:

3 $$\begin{aligned} \Theta _{(i,j)}(k) = {\mathbb {F}}\left\{ P_{(i,j)}(k)\right\} , \end{aligned}$$

where ${\mathbb {F}}$ is the FM transformation of pixel $P_{(i,j)}(k)$ that results in the focus value $\Theta _{(i,j)}$ in the $kth$ image. It represents the focus behavior or the focus curve of the pixel^33^.

In conventional methods, the initial depth map $D_{(i,j)}$ is obtained by maximizing the focus curve along the optical axis^9,34–37^. The value of *k* is obtained using (4), where $\Theta _{(i,j)}(k)$ is at the maximum according to:

4 $$\begin{aligned} D_{(i,j)} = \underset{k}{\arg \max } (\Theta _{(i,j)}(k)). \end{aligned}$$

However, the obtain depth map can be further improved by some depth adjustment using the correction suggested by Shim^38^:

5 $$\begin{aligned} D_{(i,j)}^{corrected} = \frac{f\times D_{(i,j)}}{D_{(i,j)} - f}, \end{aligned}$$

where *f* denotes the focal length of the imaging device.

The focus behavior of every individual pixel, or in other words the focus curves, depends on various factors such as the type of FM used, the camera parameters, and most importantly, the image texture around that object point^9,13,39^. If the images are properly acquired, then all the resulting focus curves are bell-shaped^16^. The Gaussian model^15,29^, Lorentz-Cauchy model^9^, and Quadratic model^40^ are used to approximate these bell-shaped focus curves^41^.

The Gaussian model^42^ is given by:

6 $$\begin{aligned} \Theta _G(k)=A_G\exp \left[ -\frac{(k-B_G)^2}{2C_G ^2}\right] , \end{aligned}$$

the Lorentz-Cauchy model is given by:

7 $$\begin{aligned} \Theta _L(k)=A_L \frac{B_L ^2}{B_L ^2 + (k-C_L)^2}, \end{aligned}$$

and the Quadratic model is given by:

8 $$\begin{aligned} \Theta _Q(k)=A_Q k^2 + B_Q k + C_Q, \end{aligned}$$

where the *A*s, *B*s and *C*s are the parameters of the respective model. The unification of these models in the Quadratic model is provided by^41^. If (6) is simplified after logarithmic transformation, it transforms to (8) as follows:

9 $$\begin{aligned} \begin{aligned} \Theta _G&\rightarrow \Theta _Q,\\ \log (\Theta _G(k))&=\log (A_G) -\frac{1}{2C_G ^2}(k^2 + B_G^2 - 2B_G k). \end{aligned} \end{aligned}$$

Equation (7) can be transformed to (8) by applying a reciprocal transformation and some simplification, as follows:

10 $$\begin{aligned} \begin{aligned} \Theta _L&\rightarrow \Theta _Q,\\ \frac{1}{\Theta _L(k)}&= \frac{1}{A_L B_L ^2} (B_L ^2 +k^2 +C_L ^2 - 2C_L k). \end{aligned} \end{aligned}$$

**Figure 3:**
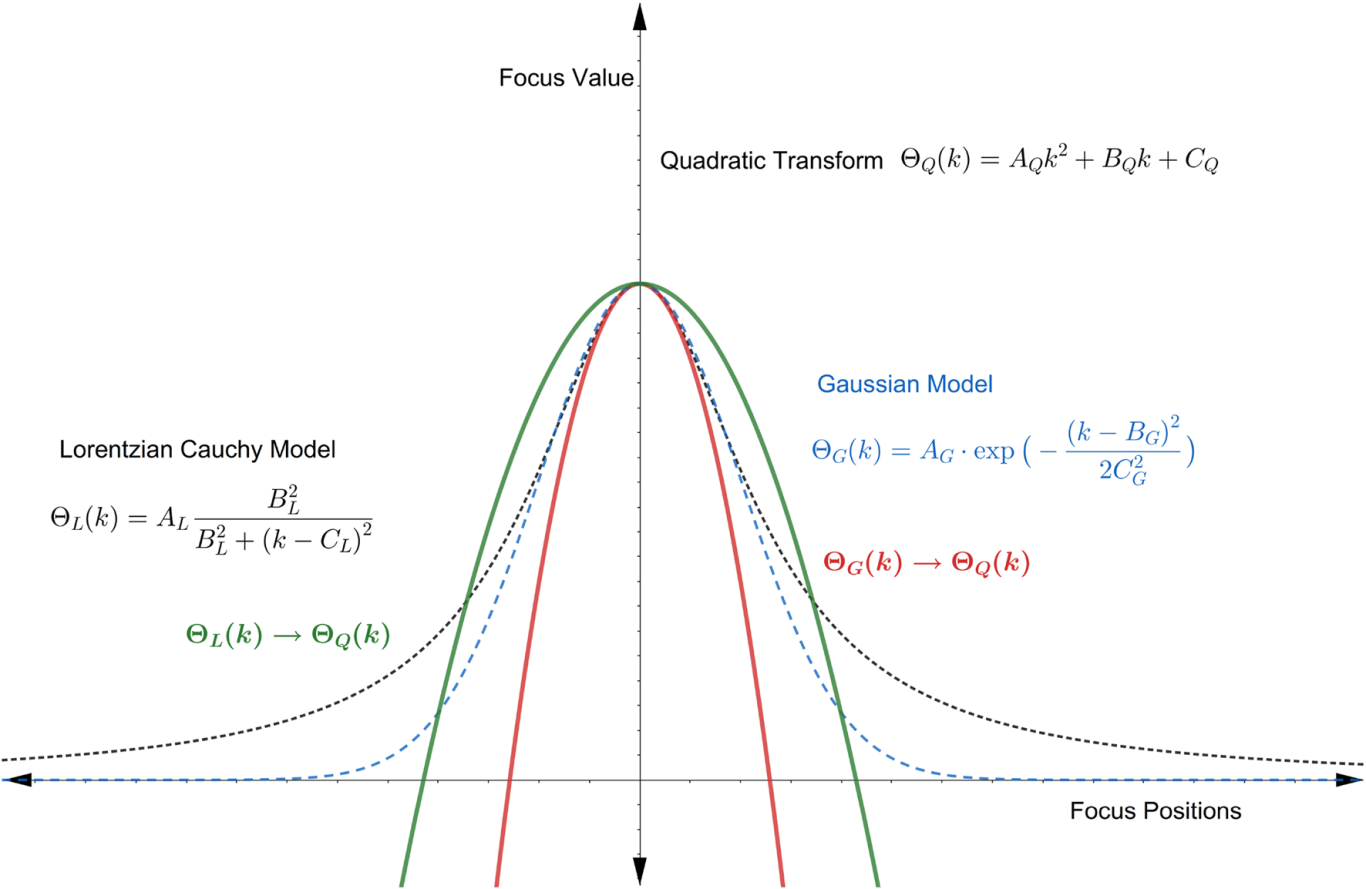
Transformation of Gaussian and Lorentz-Cauchy models into Quadratic model.

The transformation of the Gaussian and Lorentz-Cauchy models to the Quadratic model is shown in Fig. 3. In the next section, the Quadratic model as given by (8) is used to model the Jitter noise in SFF systems.

## Motivation

Shape from focus has been under research for many years, but there exist some unresolved problems that impact the system performance. One of them is the instability in the sampling step size. The images are meant to be obtained at different focus levels, at a predetermined constant step size^9^. However, in practice, this step size cannot remain constant because of the mechanical aspects of the imaging device and lens-focusing methods. The instability in sampling step size is referred to as Jittering or Jitter noise. This noise alters the focus values of the image, by oscillating along the optical axis and propagates through the entire stack^33,43^. Since the same level of error is present in all the images, it is impossible to eliminate it through traditional de-noising techniques.

Jang et al.^44^ used the Kalman filter to remove this noise. They also proposed other methods involving Kalman filter^43,45–48^. However, all these methods used Kalman filter with scalar models. The system matrix was considered as 1, hence ignoring the dynamic nature of the focus cues. All of their methods obtained multiple images for eliminating jitter at each step. It means if there were *n* images in the stack, 100 samples for each step were obtained, thus requiring $n\times 100$ samples for each focus curve. However, similar results can be obtained by taking the mean of the measurement values on every step position *k*. Also, the large number of images increases the complexity of the system, resulting in high computational costs, impacting the practical use of their methods. Jitter noise is assumed to have Normal (symmetric) and Levy distributions (non-symmetric) with fixed parameters^44,47^. Later, a dynamic Kalman filter was used to address these issues proving that the resultant Jitter noise is dynamic in nature and is position-dependent^33^. Its probability density function changes on every step, and depends on many factors.

In this manuscript, the modeling of Jitter noise is presented in section "Focus measurement and focus curve modeling", and the recursive least squares (RLS) filter is designed to eliminate this noise from the SFF systems. In this method, a single new data point is analyzed in each algorithm iteration in order to improve the estimation of the model parameters. Since RLS converges faster, an RLS filter is designed to remove the Jitter noise from the focus curves. The Quadratic function is used to design the measurement model of focus curves, and an expression for shape recovery is followed. In the proposed scheme, a single measurement is taken at each step. Thus, as opposed to previously proposed methods, only *n* samples are required for each focus curve.

The manuscript organization is as follows. Section “Proposed method” discusses Jitter noise modeling and the proposed methodology of this noise remval using RLS. Section “Results and discussions” presents the results and discussions. Finally, Section “Conclusion” presents the conclusion of this study.

## Proposed method

Jittering or Jitter noise is an error in signal amplitude, caused by the variation in sampling frequency while sampling a signal. In SFF systems, Jitter occurs when there is uncertainty or unevenness in the sampling step size of either *u* or *f*, depending on the variable factor to acquire the stack of images. This section discusses the step size in both situations of image acquisition, followed by modeling of Jitter noise which will be later utilized by the proposed model.

### Conditions and assumptions for SFF

There are four main conditions for image acquisition in Shape from Focus systems. First, the object is moved towards (or away) from the lens, ensuring that the whole object is first defocused then it is gradually focused (on every point) and then completely defocused again^34^. This condition ensures that the object only moves along the optical axis and there is no movement perpendicular to the optical axis. Second, as the object is moved, the magnification of the imaging system is assumed to be constant for the image areas that are perfectly focused^8^. Third, the body (object) is piece-wise constant, and there is no occlusion in the scene^36^. Last, the Depth of Field (*DoF*) of the imaging device in SFF systems must be finite and shallow (as much as possible)^9^. If any of these three conditions is violated then SFF algorithms can not be applied unless pre-processing of the image-stack is performed, e.g. image registration, image resize, etc.

The first condition ensures that complete bell-shaped focus curves are obtained. If this condition is violated, then obtaining complete bell-shaped focus curves becomes difficult or impossible. If the *DoF* is infinite or large, then the acquired images will have the same degree of focus, voilating the second condition, thus making it difficult to measure depth/shape using image focus. The shallow *DoF* also guarantees that the magnification for focused points in the image-stack, is not changed or the change is minimal.

### Step size in SFF image acquisition

Observing all the necessary conditions of SFF mentioned above, i.e. the object is moved towards or away from the lens in constant small steps of $\Delta u$, the depth of field is as shallow as possible, and the focal length as well as magnification are kept constant; the step size expression *u* is provided by Muhammad and Choi^9^. The simplified expression for $\Delta u$ (step size) can be given as:

11 $$\begin{aligned} \Delta u = \frac{DoF}{12}, \end{aligned}$$

which is the maximum limit for $\Delta u$^9^.

As mentioned before, the images can also be obtained by changing the focal length of the system in small, constant increments of $\Delta f$^49^. In this case the focal length of the device is varied while the object is held static in front of the imaging device. This type of technique is mostly used by auto-focusing algorithms, for searching the best focal position for a single point. It can also be used for depth and shape estimation of the object under consideration^11^. Regardless of the image acquisition technique, an image is stored at every step to obtain a stack of images.

### Modeling Jitter in SFF

This paper utilizes Quadratic function to model the jitter:

12 $$\begin{aligned} f(k) = a k^2+bk+c, \end{aligned}$$

where *a*, *b* and *c* are the function parameters, and *f*(*k*) is the Quadratic function. The sample points of this function are represented by $1\le k\le n$. The step size is $\Delta k$, and is 1. For modeling Jitter noise, the uncertainty in step size is considered as, $\zeta \sim N(0, \sigma ^2 _\zeta )$ and thus (12) can be written as follows:

13 $$\begin{aligned} f(k+\zeta ) = a(k+\zeta )^2+b(k+\zeta )+c. \end{aligned}$$

By expanding the squared terms and simplifying using the Taylor series^50^ , (13) can be written as:

14 $$\begin{aligned} f(k+\zeta ) = ak^2+bk+c +\zeta (2ak + b) + \zeta ^2 a. \end{aligned}$$

the following equation is obtained by using (12) and (14):

15 $$\begin{aligned} f(k+\zeta ) = f(k) +\zeta f'(k) + \zeta ^2 \frac{f''(k)}{2}. \end{aligned}$$

It can be observed in (15) that the noise on the RHS of the equation is multiplied by the first and second derivatives of the function. Therefore, it can be concluded that the Jitter noise, in SFF systems, is dependent on the slope and concavity of the focus curves. If $\zeta$ is Normal ($N(0,\sigma _{\zeta }^2)$), then ($k+\zeta$) follows $N(k,\sigma _{\zeta }^2)$, which given by:

16 $$\begin{aligned} p(\zeta ) = \frac{1}{\sigma _\zeta \sqrt{2\pi }}\exp \left[ {-\frac{1}{2}\big (\frac{\zeta -k}{\sigma _\zeta }\big )^2}\right] . \end{aligned}$$

Rewriting (15):

17 $$\begin{aligned} \begin{aligned} f(k+\zeta )&= f(k) +\xi _1 + \xi _2\\&= f(k)+\xi , \end{aligned} \end{aligned}$$

where $\xi _1$ and $\xi _2$ are given by:

$$\begin{aligned} \xi _1 = \zeta f'(k) = \zeta (2ak+b), \end{aligned}$$

and,

$$\begin{aligned} \xi _2 =\zeta ^2 \frac{f''(k)}{2}= \zeta ^2a, \end{aligned}$$

where $\xi _1$ is normally distributed, *mean* $\mu _1 = 0$ and *variance* $\sigma _1^2 = (2ak+b)^2 \sigma _{\zeta }^2$.

Meanwhile, $\xi _2$ follows a Gamma ($\Gamma$) distribution, with *mean* $\mu _2=a \sigma ^2_{\zeta }$, and *variance* $\sigma _2 ^2=2a ^2 \sigma ^4_{\zeta }$. The value of the *variance* $\sigma _{1}^2$ is different at every $k{th}$ step, and becomes zero at:

$$\begin{aligned} k=-\frac{b}{2a}. \end{aligned}$$

The direction of $\xi _2$ depends on the sign of *a*, and will always tend to the concavity of the function. The range ($R(\xi )$) of Jitter noise is provided by (18):

18 $$\begin{aligned} R(\xi ) = \left\{ \begin{array}{c l} \xi \ge -\frac{(2ak+b)^2}{4a} &{} a>0,\\ \xi \le -\frac{(2ak+b)^2}{4a} &{} a<0,\\ -\infty<\xi <\infty &{}a=0. \end{array}\right. \end{aligned}$$

In order to obtain the pdf ($p(\xi )$) of the Jitter noise, $\xi _1$ and $\xi _2$ can be combined as $\xi =\xi _1 +\xi _2$, and $p(\xi )$ is given by (19), where the symbols $r_1$ and $r_2$ in (19) are computed using the following equation:

$$\begin{aligned} r_{1,2}= -\frac{\left( -(2ak+b)\pm \sqrt{(2ak+b)^2 +4a\xi }\right) ^2}{8a^2\sigma _\zeta ^2}. \end{aligned}$$

Expression (19) holds only if $\zeta$ follows normal distribution. The Jitter noise $\xi$ is the resulting noise in focus values due to uncertainty $\zeta$ in sampling step $\Delta u$.

19 $$\begin{aligned} p(\xi ) = \left\{ \begin{array}{lclr} \frac{1}{\sigma _\zeta \sqrt{2\pi }}\frac{1}{\sqrt{(2ak+b)^2 +4a\xi }} \left( \exp [r_1]+\exp [r_2]\right) &{} \forall &{} \xi \in (-\infty , -\frac{(2ak+b)^2}{4a}]\cup (-\frac{(2ak+b)^2}{4a}, \infty ),&{}a\ne 0, \\ \frac{1}{b\sigma _\zeta \sqrt{2\pi }}\exp \left[ \frac{\xi ^2}{2b^2\sigma _\zeta ^2}\right] &{}\forall &{} \xi \in (-\infty ,\infty ),&{}a=0. \end{array}\right. \end{aligned}$$

### Proposed Jitter removal methodology

The proposed scheme can be applied after the FM transformation is performed using (3). Pertaining to its faster convergence property, a recursive least squares (RLS) filter is designed to fully eliminate the Jitter noise from the focus curves. RLS is one of the most well-known adaptive filters that recursively calculate the coefficients of a given function, minimizing the cost function related to input measurements^51,52^.

#### Recursive least squares filter design

The measurements of focus curves (of every individual pixel) depend on various factors^9,13^. These focus curves are bell-shaped^16^ and hence different models such as Gaussian model^15^, Lorentz-Cauchy model^9^, and Quadratic model^40^ are used to approximate them. The advantage of the Quadratic model is, that it makes the focus profiles collected by different FMs to be modeled in a common form that is easy to handle and has computational advantages^41^. Thus, the measurement model of the proposed RLS scheme uses the Quadratic function given in (12), and can also be represented using (20):

**Table 1:** Summary of RLS filter equations for proposed scheme.

Equation name	Equation
*f*(*k*) represents	$a k^2 +b k +c$
Parameters vector	$X = [a,b,c]^T$
Measure-ment vector	$y(k) = H(k) X+\xi (k)$
Measure-ment matrix	$H(k) = [k^2,k,1]$
**Recursive least squares filter equations**	
Filter gain: *L*(*k*)	$$\Psi (k-1)H(k)^T\begin{bmatrix} H(k)\Psi (k-1) H(k)^T + \sigma _\xi ^2 \end{bmatrix}^{-1}$$
Estimate: $\hat{X}(k)$	$$\hat{X}(k-1) + L(k) \begin{bmatrix}y(k) - H(k)\hat{X}(k-1) \end{bmatrix}$$
Covariance: $\Psi (k)$	$$\begin{bmatrix}\Psi ^{-1}(k-1)+\frac{1}{\sigma _\xi ^2}H^T(k)H(k)\end{bmatrix}^{-1}$$

20 $$\begin{aligned} f(k) = \begin{bmatrix} k^2&k&1 \end{bmatrix} \begin{bmatrix} a\\ b\\ c\\ \end{bmatrix}, \end{aligned}$$

where *a*, *b*, and *c* are the unknown constant coefficients, and by measuring this function, a measurement model is obtained as:
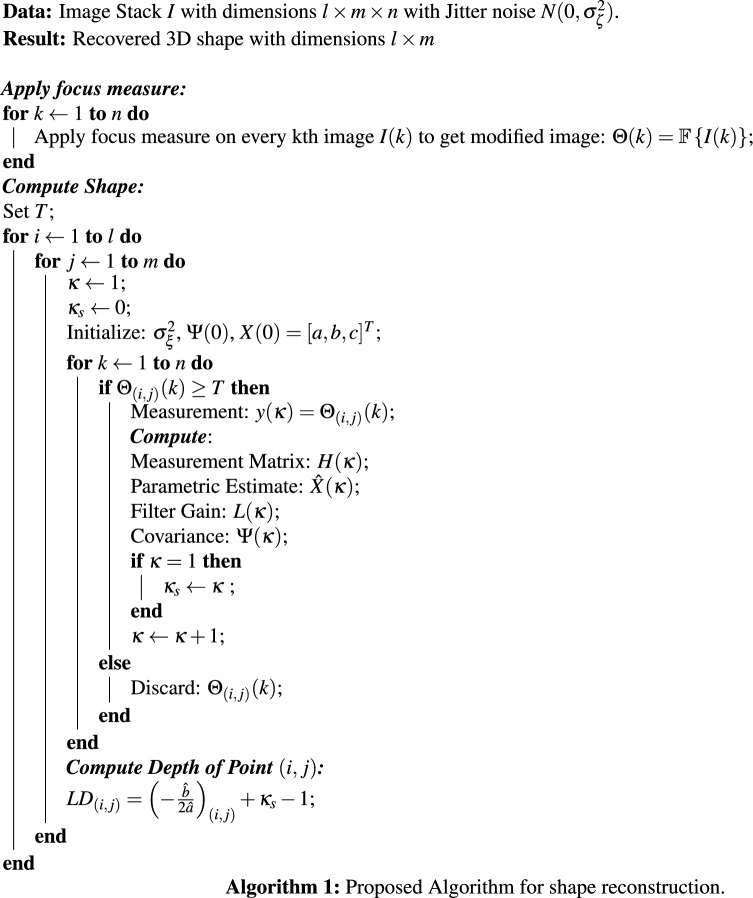

21 $$\begin{aligned} y(k) = H(k) X + \xi (k), \end{aligned}$$

where *y*(*k*) denotes the measurements of focus curve after FM application at the $kth$ image, *H*(*k*) is the measurement matrix at *k*, and $\xi (k)$ is the measurement error representing Jitter noise with pdf given in (19). The unknown parametric vector is $X=[a,b,c]^T$. The RLS gain *L*(*k*) is computed on every step *k* as follows:

22 $$\begin{aligned} L(k)= \Psi (k-1) H(k)^T \begin{bmatrix} H(k)\Psi (k-1) H(k)^T + \sigma _\xi ^2 \end{bmatrix}^{-1} , \end{aligned}$$

where $\sigma _\xi ^2=Var(\xi )$ is the variance of Jitter noise. The optimal least squares estimate of the parametric vector *X* and covariance $\Psi (k)$ are computed by the following recursive equations:

23 $$\begin{aligned} \begin{aligned} \hat{X}(k)&= \hat{X}(k-1) + L(k) \begin{bmatrix}y(k) - H(k)\hat{X}(k-1)\end{bmatrix},\\ \Psi (k)&= \begin{bmatrix}\Psi ^{-1}(k-1)+\frac{1}{\sigma _\xi ^2}H^T(k)H(k)\end{bmatrix}^{-1}. \end{aligned} \end{aligned}$$

In (23) the covariance $\Psi (k)$ can be redesigned to remove the matrix inversions using the Sherman-Morrison formula^53–56^, and can be rewritten as follows:

24 $$\begin{aligned} \Psi (k) = \Psi (k-1)-\frac{1}{\sigma _\xi ^2 + H\Psi (k-1)H^T}\Psi (k-1)H^T(k)H(k)\Psi (k-1). \end{aligned}$$

The summary of equations for the proposed filter design is provided in Table 1.

#### Shape recovery

At every $kth$ iteration, the values of $X = [a,b,c]^T$ are estimated and the depth for each pixel is updated using following equation:

25 $$\begin{aligned} LD_{(i,j)} = \left( -\frac{\hat{b}}{2\hat{a}}\right) _{(i,j)}, \end{aligned}$$

with maximum focus values being given by:

26 $$\begin{aligned} L\Theta _{(i,j)} = \left( -\frac{{\hat{b}}^2}{4\hat{a}} +c\right) _{(i,j)}. \end{aligned}$$

#### Proposed algorithm

The proposed algorithm is listed in Algorithm 1. First, the FM is applied on every image of the image stack (*I*) to obtain the modified image stack ($\Theta$) as follows:

27 $$\begin{aligned} \Theta (k) = {\mathbb {F}}\left\{ I(k)\right\} . \end{aligned}$$

After the modified stack is obtained, the values of the focus curve ($\Theta _{(i,j)}(k)$) for each object point are taken and compared to a threshold value (*T*) (which is set heuristically to ignore tails of the focus curves). The values of $\Theta _{(i,j)}(k)<T$ are ignored. Additionally, for values of $\Theta _{(i,j)}(k)\ge T$, the value of index *k* is stored in $\kappa _s$, followed by initialization of a new index parameter $\kappa$. The measurement value of focus for point (*i*, *j*) in consideration is taken as $y(\kappa) =\Theta _{(i,j)}(k)$. The next step calculates state measurement matrix ($H(\kappa )$), parametric vector ($\hat{X}(\kappa )$), covariance matrix ($\Psi (\kappa )$), and filter gain ($L(\kappa )$) using equations summarized in Table 1. After completion of all the iterations, the depth of the object point (*i*, *j*) is computed by modifying (25) as follows:

28 $$\begin{aligned} LD_{(i,j)} = \left( -\frac{\hat{b}}{2\hat{a}}\right) _{(i,j)}+ \kappa _s -1. \end{aligned}$$

## Results and discussions

The experimental results and discussions are presented in this section. The section is divided into three subsections. First, the experimental setup details are given, followed by the reconstructed-shape assessment criteria, and later a detailed analysis of the effects of Jitter noise and its removal using RLS filter for SFF is provided.

### Experimental setup

The experiments are performed on *seven* objects for shape reconstruction analysis. A summary of the objects used in the 3D shape analysis is provided in Table 2. *Ten* simulated datasets of *simulated cone* are generated with different focus positions and Jitter noise levels using a camera simulation software (AVS). The details of AVS are provided in^13,37,57^. The MATLAB code used to generate the simulated object image set is downloaded from^13^. The AVS software is inputted with the depth map, texture image, and camera parameters. The texture map comprises concentric circles with alternating black and white stripes. The depth maps and the texture images used for image generation via AVS for all sequences, of Simulated Cone are similar. The difference in each dataset is the uncertainty $\zeta \thicksim N(0,\sigma _\zeta ^2)$ in step size $\Delta u$ used to generate the sequences to study the effect of Jitter on shape reconstruction. The values of noise $\zeta$ with variance $\sigma _\zeta ^2$ for $\Delta u$ are 0, 0.1, 0.25, 0.5, 0.75, 1.0, 1.25, 1.5, 1.75, and 2.0, which result in Jitter noise $\xi$ with variance $\sigma _\xi ^2 = 2a^2\sigma _\zeta ^4$.

**Table 2:** Summary of experimented objects.

Name	Object Type	Image dimensions	Number of images
Simulated cone	Simulated	$360\times 360$	97
Real cone	Real	$200\times 200$	97
Real plane	Real	$200\times 200$	87
LCD-TFT	Real	$200\times 200$	60
Groove	Real	$200\times 200$	60
Coin	Real	$200\times 200$	68
Image-I	Real	$200\times 200$	60

**Figure 4:**
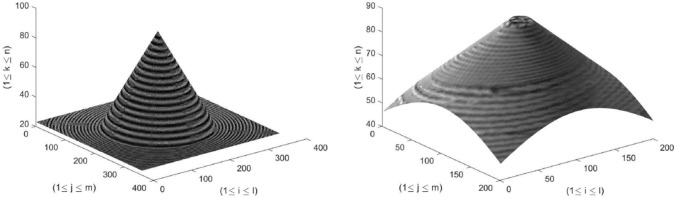
Ground truth of Simulated Cone and Real Cone.

**Figure 5:**
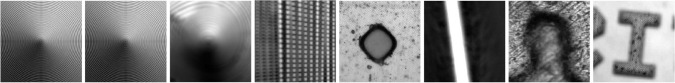
10th frame of image sequence of each experimented object. (left to right) Simulated Cone with $\sigma ^2_\xi = (2ak+b)^2 \sigma ^2_{\zeta } + 2a ^2 \sigma ^4_{\zeta }$, where $\sigma _\zeta ^2 = 0$ and $\sigma _\zeta ^2 = 2.0$, Real Cone, Real Plane, LCD-TFT, Groove, Coin and Image-I.

The *real* datasets consist of real objects, Real Cone, Real Plane, LCD-TFT Filter, Groove, Coin, and Image-I. These image sequences were originally in grayscale. Figure 4 provides the ground truths of the Simulated and Real cones. Figure 5 shows the 10th frame of each image sequence. These image sequences have been widely used by many researchers including^11,24,40,58–60^.

Images of Real Cone and Real Plane were taken using the CCD camera system^17^. The LCD-TFT filter images are microscopic images of an LCD color filter. The coin sequence consists of magnified images of Lincoln’s head from the back of the US penny. The sequence of Image-I is the letter *I* engraved in a metallic surface. These images were obtained using the microscopic control system (MCS). This system comprised of a personal computer integrated with a frame grabber board (Matrox Meteor-II) and a CCD camera (SAMSUNG CAMERA SCC-341) mounted on a microscope (NIKON OPTIPHOT-100S). Computer software in MCS acquired the images by controlling the lens position through a stepper motor driver (MAC 5,000), possessing a 2.5*nm* minimum step length. Every image stored for each sequence at every step was captured by varying the object plane.

The Groove image sequence consists of a V-groove engraved in a metallic surface.

### Metric measures

**Table 3:** Comparison of proposed method with previous methods using SML, GLVA and TENG with Jitter Noise ($\sigma ^2_\xi = (2ak+b)^2 \sigma ^2_{\zeta } + 2a ^2 \sigma ^4_{\zeta }$), $\sigma ^2_\zeta = 0.1$.

Methods	SML			GLVA			TENG			Iterations
	RMSE	Cor.	DD	RMSE	Cor.	DD	RMSE	Cor.	DD	
ScalarKF	10.353	0.687	3.755	10.989	0.681	4.029	9.258	0.805	3.054	100
MKF	9.215	0.731	5.969	8.006	0.825	5.182	7.298	0.873	5.565	100
ANNF	5.620	0.950	5.399	5.288	0.924	1.996	6.347	0.879	1.859	100
MCCKF	5.554	0.949	4.363	5.244	0.924	1.673	6.337	0.880	1.815	100
IMCCKF	5.333	0.949	4.404	5.208	0.925	1.314	6.338	0.879	1.815	100
Dynamic KF^33^	5.279	0.948	1.253	5.158	0.938	1.287	4.800	0.946	1.532	1
Proposed RLS method	4.796	0.953	0.738	4.861	0.948	0.964	4.907	0.951	1.015	1

The quality of shape reconstruction can be measured by the difference between the reconstructed shape and the ideal shape, and it is set to be degraded when the difference is increased. This section analyzes the quality of the depth map obtained by using different FMs under various levels of Jitter noise. The quality of depth map is maximum when the obtained depth map is indistinguishable from the original map, and the difference is zero. In the previous literature, several quality metrics have been presented for accessing the quality of shape^61^. In this manuscript, RMSE and correlation are used to compare the proposed method combined with various FM operators under different levels of Jitter noise. A new depth assessment metric referred to as depth distortion (DD) is also proposed in this manuscript.

*Root Mean Square Error* (RMSE) is the square root of the variance of the residuals of the data under observation. This indicates how close the perceived shape is to the original shape^62^, and is given by:

29 $$\begin{aligned} RMSE = \sqrt{\frac{1}{(l\times m)}\sum _i^l\sum _j^m\left( G^{true}_{(i,j)} - D^{obtained}_{(i,j)}\right) ^2} \end{aligned}$$

where $G^{true}_{(i,j)}$ is the ground truth, $D^{obtained}_{(i,j)}$ is the obtained depth map, and $l\times m$ are the dimensions of the depth maps. A higher value of *RMSE* indicates a larger error in the shape reconstruction. For better results, the value of RMSE should be closer to zero.

*Correlation* is a similarity measure between two shapes^62^, and is given by:

30 $$\begin{aligned} \rho_{GD} = \frac{cov(G^{true},D^{obtained})}{\sigma_G \sigma_D} \end{aligned}$$

where *cov* is the covariance, and $\sigma _G^2$ and $\sigma _D^2$ are the variances of $G^{true}$ and $D^{obtained}$, respectively.

*Depth Distortion* (DD) metric indicates the number of pixels (in percentage), which contribute to RMSE. It is calculated as follows:

31 $$\begin{aligned} DD_{GD} = \left( \frac{|\left( \sum _{l\times m}{E_G}\right) - \left( \sum _{l\times m}E_D\right) |}{l\times m}\right) \times 100, \end{aligned}$$

where, $E_G$ and $E_D$ are the edge images of the ground truth and the obtained depth map, respectively, and can be calculated by applying edge operators (e.g., Canny or Sobel operator). If the ground truth has any edge, then the number of pixels contributing to that edge should be similar to the obtained depth map; thus, the difference will be zero. But if the obtained depth map has some distortions in depth, then the edge image will have extra pixels from depth distortion; hence, it will contribute to RMSE. The proposed metric determines the quantity of these pixels.

### Experimental results and discussion

Six well-known FMs–Sum of Modified Laplacian (SML), Tenenbaum (TENG), Grey Level Variance (GLVA), Image Contrast (CONT), 3D Laplacian (GRA3), and Spatial Frequency (SFRQ) are used for the current experiments.

The noise $\zeta \sim N(0,\sigma _\zeta ^2)$ is simulated with different values of variance $\sigma _\zeta ^2$ in $\Delta u$, generating Jitter noise having variance $\sigma ^2_\xi = (2ak+b)^2 \sigma ^2_{\zeta } + 2a ^2 \sigma ^4_{\zeta }$. The simulation values for variance $\sigma _\zeta ^2$ in the experiments are taken as 0, 0.1, 0.25, 0.5, 0.75, 1.0, 1.25, 1.5, 1.75, and 2.0. The experiments are first performed on the Simulated Cone image sequence by simulating this noise for all the six above-mentioned FMs. Tables 4, 5, 6, 7, 8, 9 show the results of the reconstructed shape of Simulated Cone with Jitter noise with variance $\sigma ^2_\xi$, where $0\le \sigma _\zeta ^2 \le 2$.

In Table 3, the proposed method is compared with previous methods provided. The methods include Scalar KF^43,44^, Modified Kalman Filter^47^, Adaptive Neural Network Filter^45^, Maximum Correntropy Criterion^48^ and Improved Maximum Correntropy Criterion Kalman Filter^48^; with $\sigma ^2_\zeta = 0.1$. All these methods use 100 iterations per step, whereas, the proposed scheme utilizes only a single measurement for each step. RMSE, correlation and DD results listed in the table utilize SML, GLVA, and TENG FMs. The results of proposed technique in terms all the three measures have outclassed every single of the previous methods.

Along with the above mentioned methods, the proposed RLS method is also compared with the dynamic Kalman filter as proposed by Mutahira et al.^33^, which also utilizes just one iteration per step. However, since the system model in their method is modeled using Taylor’s series, it first estimates the derivatives of the function ($f'(k), f''(k) \& f'''(k)$) using a single measurement of *f*(*k*) and then using these estimates (of the derivatives), the parameters of the cubic equation (*a*, *b*, *c*, and *d*) are estimated. Later the depth is recovered using the indirect estimated values of the parameters. This indirect estimation introduces lags and errors in the final depth map. The proposed scheme presented in this manuscript is based on estimating the parameters of the quadratic equation (*a*, *b*, and *c*) directly from the measurement. Thus, depth estimation using proposed scheme produces significantly lower error contributing to better RMSE, DD and correlation values, as can be seen in Table 3.

**Table 4:** RMSE, Correlation (Cor.) and Depth Distortion (DD) in % for shape reconstruction using FM:SML with different levels of variance of Jitter noise ($\sigma ^2_\xi = (2ak+b)^2 \sigma ^2_{\zeta } + 2a ^2 \sigma ^4_{\zeta }$), with and without proposed RLS filter.

$\sigma _\zeta ^2$	Cor.		RMSE		DD	
	With FM only	With RLS & FM	With FM only	With RLS & FM	With FM only	With RLS & FM
0	0.952	0.952	4.855	4.811	2.668	0.445
0.1	0.953	0.953	4.830	4.796	2.691	0.738
0.25	0.952	0.952	4.880	4.833	2.894	1.140
0.5	0.952	0.953	4.833	4.835	4.617	0.824
0.75	0.951	0.952	4.832	4.778	4.725	1.603
1	0.940	0.951	5.179	4.821	4.502	2.613
1.25	0.949	0.953	5.261	4.848	3.859	2.244
1.5	0.929	0.949	5.425	4.928	5.413	2.508
1.75	0.938	0.946	5.990	5.012	5.237	2.633
2	0.931	0.949	5.880	5.035	5.004	1.616
Av.	0.945	0.951	5.197	4.870	4.161	1.636

**Table 5:** RMSE, Correlation (Cor.) and Depth Distortion (DD) in % for shape reconstruction using FM:CONT with different levels of variance of Jitter noise ($\sigma ^2_\xi = (2ak+b)^2 \sigma ^2_{\zeta } + 2a ^2 \sigma ^4_{\zeta }$), with and without proposed RLS filter.

$\sigma _\zeta ^2$	Cor.		RMSE		DD	
	With FM only	With RLS & FM	With FM only	With RLS & FM	With FM only	With RLS & FM
0	0.947	0.948	4.991	4.936	2.084	0.640
0.1	0.946	0.948	4.992	4.933	2.192	0.616
0.25	0.947	0.947	5.013	4.966	2.139	0.646
0.5	0.947	0.949	4.966	4.941	2.198	0.632
0.75	0.947	0.948	5.006	4.987	2.167	0.777
1	0.937	0.949	5.218	4.887	1.998	0.664
1.25	0.941	0.945	5.265	4.986	2.235	1.015
1.5	0.931	0.947	5.346	4.998	2.737	1.894
1.75	0.939	0.949	5.183	4.939	3.215	2.306
2	0.934	0.949	5.151	4.924	2.532	0.985
Av.	0.942	0.948	5.113	4.950	2.350	1.018

**Table 6:** RMSE, Correlation (Cor.) and Depth Distortion (DD) in % for shape reconstruction using FM:GLVA with different levels of variance of Jitter noise ($\sigma ^2_\xi = (2ak+b)^2 \sigma ^2_{\zeta } + 2a ^2 \sigma ^4_{\zeta }$), with and without proposed RLS filter.

$\sigma _\zeta ^2$	Cor.		RMSE		DD	
	With FM only	With RLS & FM	With FM only	With RLS & FM	With FM only	With RLS & FM
0	0.951	0.951	4.879	4.872	2.495	1.009
0.1	0.951	0.952	4.872	4.861	2.504	0.964
0.25	0.952	0.952	4.870	4.866	3.017	1.142
0.5	0.951	0.952	4.892	4.859	4.590	1.681
0.75	0.951	0.951	4.860	4.856	4.663	2.135
1	0.941	0.953	5.170	4.785	3.026	2.243
1.25	0.950	0.952	4.950	4.970	3.787	1.729
1.5	0.928	0.951	5.434	4.882	5.391	2.601
1.75	0.939	0.948	5.259	5.052	3.367	2.552
2	0.931	0.945	5.370	5.090	4.270	2.983
Av.	0.944	0.951	5.055	4.909	3.711	1.904

**Table 7:** RMSE, Correlation (Cor.) and Depth Distortion (DD) in % for shape reconstruction using FM:GRA3 with different levels of variance of Jitter noise ($\sigma ^2_\xi = (2ak+b)^2 \sigma ^2_{\zeta } + 2a ^2 \sigma ^4_{\zeta }$), with and without proposed RLS filter.

$\sigma _\zeta ^2$	Cor.		RMSE		DD	
	With FM only	With RLS & FM	With FM only	With RLS & FM	With FM only	With RLS & FM
0	0.950	0.9501	4.924	4.9221	4.017	0.459
0.1	0.950	0.950	4.909	4.926	3.664	0.451
0.25	0.950	0.949	4.918	4.950	3.253	0.410
0.5	0.948	0.951	4.963	4.868	4.163	0.573
0.75	0.952	0.952	4.894	4.831	4.201	1.368
1	0.938	0.951	5.224	4.822	3.211	1.166
1.25	0.949	0.950	4.955	4.923	3.796	1.600
1.5	0.930	0.949	5.388	4.941	4.713	2.405
1.75	0.940	0.950	5.248	4.980	3.971	1.949
2	0.943	0.946	5.322	5.015	3.971	1.617
Av.	0.945	0.950	5.075	4.918	3.896	1.200

**Table 8:** RMSE, Correlation (Cor.) and Depth Distortion (DD) in % for shape reconstruction using FM:SFRQ with different levels of variance of Jitter noise ($\sigma ^2_\xi = (2ak+b)^2 \sigma ^2_{\zeta } + 2a ^2 \sigma ^4_{\zeta }$), with and without proposed RLS filter.

$\sigma _\zeta ^2$	Cor.		RMSE		DD	
	With FM only	With RLS & FM	With FM only	With RLS & FM	With FM only	With RLS & FM
0	0.947	0.947	5.006	4.957	0.617	0.441
0.1	0.947	0.948	5.009	4.946	0.582	0.432
0.25	0.946	0.947	5.033	4.990	0.651	0.421
0.5	0.949	0.948	4.935	4.919	0.927	0.422
0.75	0.949	0.945	4.994	4.866	1.093	0.465
1	0.937	0.948	5.269	4.917	1.814	0.457
1.25	0.945	0.946	5.117	5.006	1.283	0.562
1.5	0.926	0.946	5.486	5.015	2.450	1.470
1.75	0.934	0.947	5.355	5.009	1.593	1.002
2	0.939	0.943	5.241	5.098	1.868	0.687
Av.	0.942	0.947	5.145	4.972	1.288	0.636

**Table 9:** RMSE, Correlation (Cor.) and Depth Distortion (DD) in % for shape reconstruction using FM:TENG with different levels of variance of Jitter noise ($\sigma ^2_\xi = (2ak+b)^2 \sigma ^2_{\zeta } + 2a ^2 \sigma ^4_{\zeta }$), with and without proposed RLS filter.

	Cor.		RMSE		DD	
$\sigma _\zeta ^2$	With FM only	With RLS & FM	With FM only	With RLS & FM	With FM only	With RLS & FM
0	0.951	0.950	4.894	4.917	2.470	1.051
0.1	0.951	0.951	4.883	4.907	2.360	1.015
0.25	0.951	0.950	4.890	4.929	2.948	1.170
0.5	0.949	0.951	4.922	4.864	4.361	1.840
0.75	0.953	0.949	4.760	4.903	4.527	2.006
1	0.940	0.953	5.173	4.782	2.948	1.637
1.25	0.950	0.952	4.953	4.964	3.978	1.782
1.5	0.928	0.950	5.429	4.895	4.943	2.800
1.75	0.940	0.948	5.244	5.053	3.503	2.658
2	0.942	0.944	5.052	5.104	4.095	2.548
Av.	0.945	0.950	5.020	4.932	3.613	1.851

**Figure 6:**
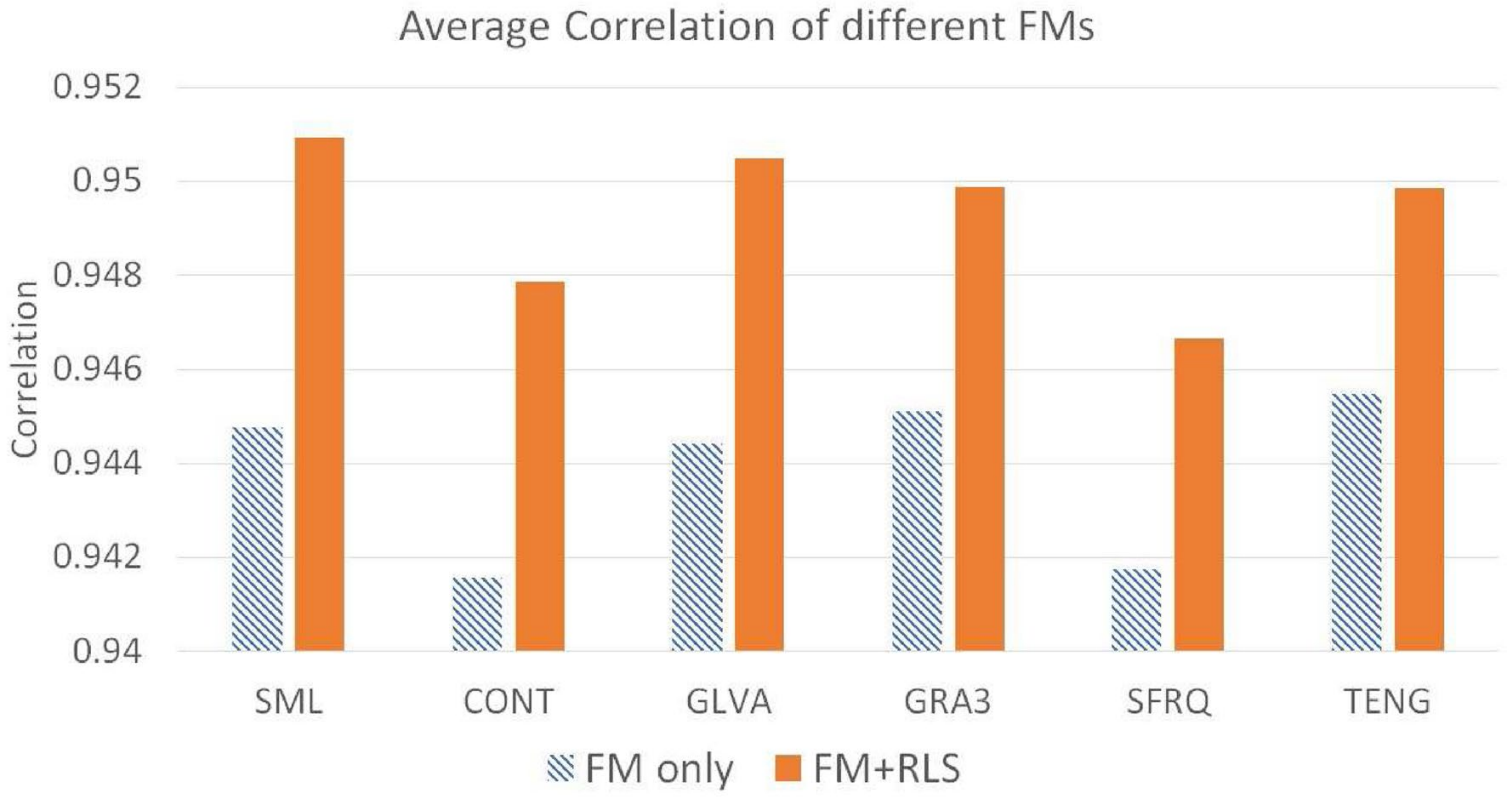
Average of Correlation for different FMs for Jitter noise $\sigma ^2_\xi = (2ak+b)^2 \sigma ^2_{\zeta } + 2a ^2 \sigma ^4_{\zeta }$ with $0\le \sigma _\zeta ^2 \le 2$.

**Figure 7:**
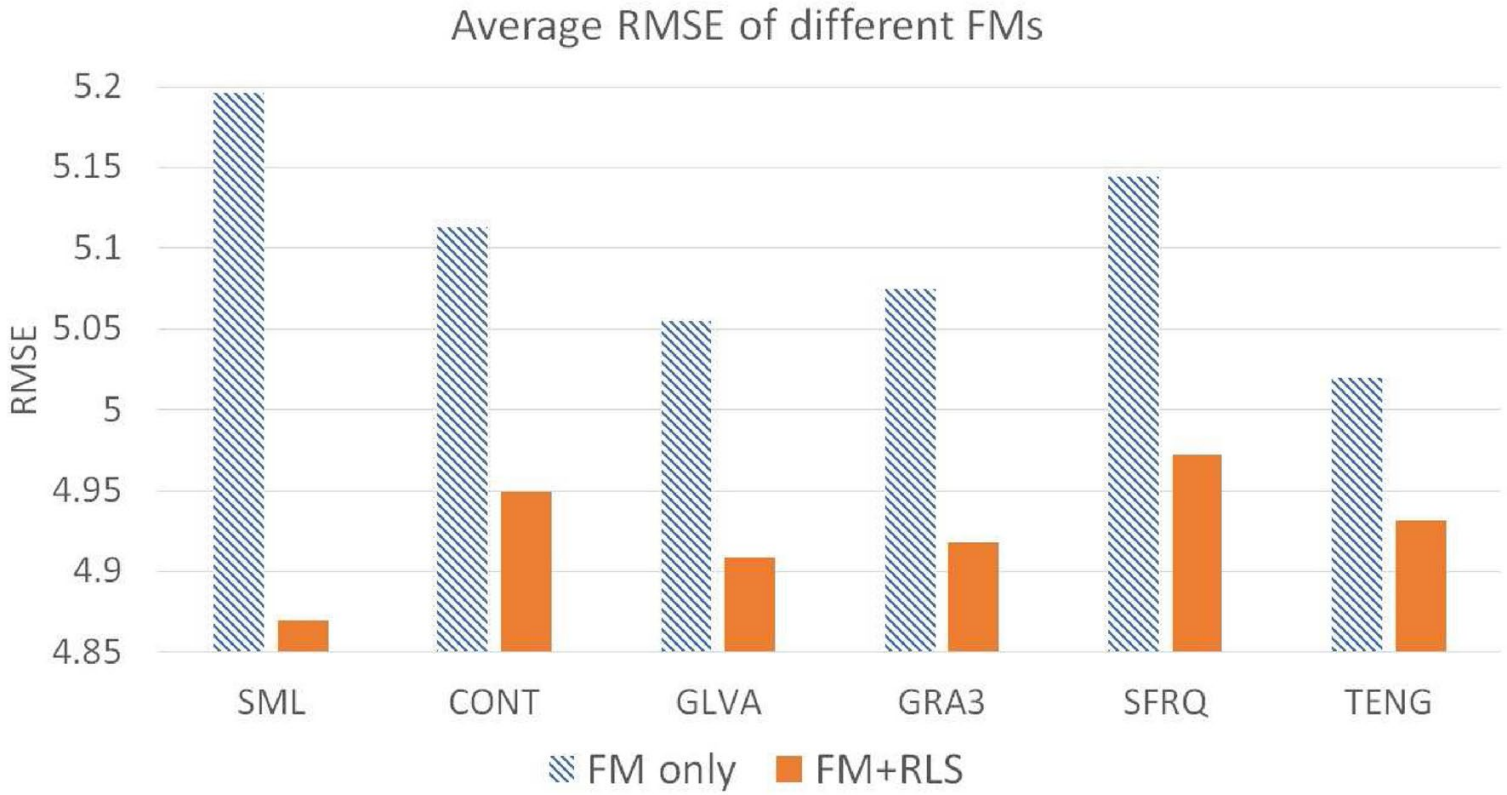
Average of RMSE for different FMs for Jitter noise $\sigma ^2_\xi = (2ak+b)^2 \sigma ^2_{\zeta } + 2a ^2 \sigma ^4_{\zeta }$ with $0\le \sigma _\zeta ^2 \le 2$.

**Figure 8:**
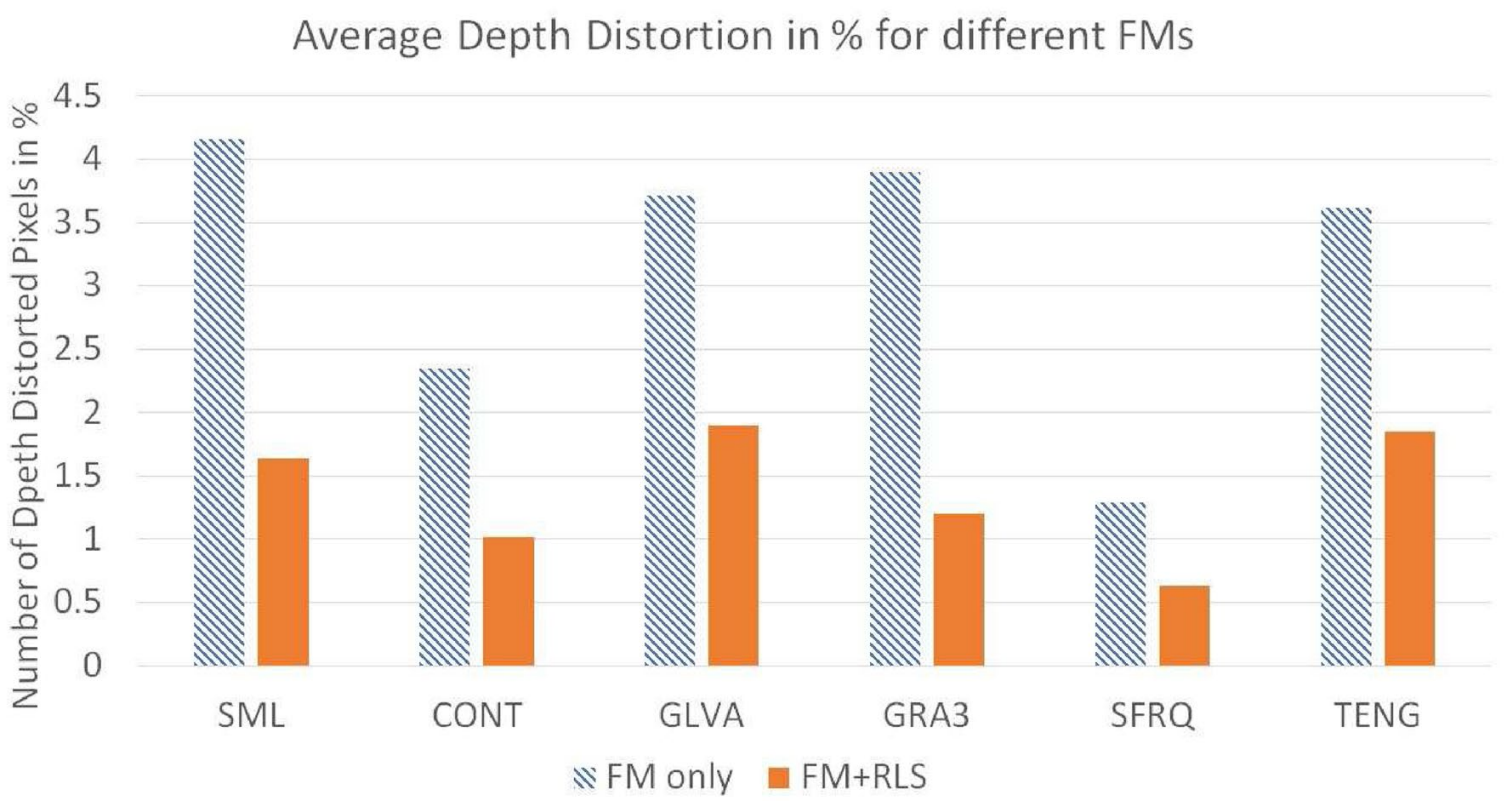
Average of Depth Distortion Metrics for different FMs for Jitter noise $\sigma ^2_\xi = (2ak+b)^2 \sigma ^2_{\zeta } + 2a ^2 \sigma ^4_{\zeta }$ with $0\le \sigma _\zeta ^2 \le 2$.

Tables 4, 5, 6, 7, 8, 9 represent the RMSE, Correlation, and Depth Distortion metrics results, for different FMs and the same FMs used with the proposed RLS scheme. The last row of each table represents the average values for RMSE, Correlation, and Depth Distortion metrics for all noise ranges. Figures 6, 7, 8 represent the graphs of the average of shape comparison metrics for all the FMs used. The shaded bars in the graphs represent the average of metrics when FM is used only for Jitter noise with variance $\sigma ^2_\xi$, where $0\le \sigma _\zeta ^2 \le 2$. The solid bars in the graphs represent the average of metrics when FM is used with the proposed RLS technique. It can be seen that SML has shown the best results when used with the proposed RLS method. The tables and figures clearly show that when the proposed technique is used for all levels of Jitter noise the shape reconstruction results improve as compared to when using FM only.

**Figure 9:**
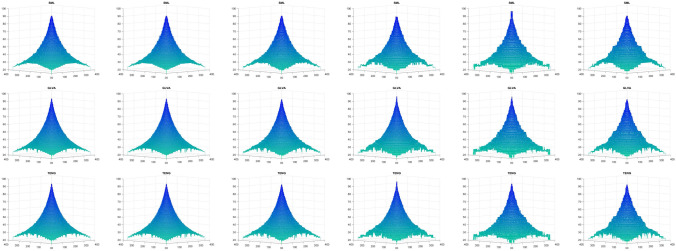
Shape reconstruction for simulated cone, using FM with proposed RLS method. (left to right) SML, GLVA, TENG. (top to bottom) $\sigma ^2_\xi = (2ak+b)^2 \sigma ^2_{\zeta } + 2a ^2 \sigma ^4_{\zeta }$ with ($\sigma _\zeta ^2 : 0, 0.1, 0.5, 1.0, 1.5, 2.0$).

**Figure 10:**
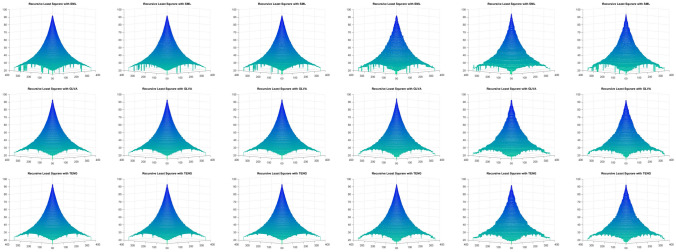
Shape reconstruction for simulated cone, using FM with proposed RLS method. (left to right) SML, GLVA, TENG. (top to bottom) $\sigma ^2_\xi = (2ak+b)^2 \sigma ^2_{\zeta } + 2a ^2 \sigma ^4_{\zeta }$ with ($\sigma _\zeta ^2 : 0, 0.1, 0.5, 1.0, 1.5, 2.0$).

Figure 9 shows the shape reconstruction of the simulated cone, when only FMs are used, and Fig. 10 represents the reconstructed shape of simulated cone when FMs are used with the proposed RLS scheme, for a noise range of $\sigma _\zeta ^2 : 0, 0.1, 0.5, 1.0, 1.5, 2.0$, respectively, which results in Jitter noise with $\sigma ^2_\xi$. These results clearly demonstrate the effectiveness of the proposed scheme. The shape reconstruction, when only FMs are used, have rough surface when the noise is increased from 0 to 2.0; whereas, for the same noise range, the proposed scheme has shown promising results.

**Figure 11:**
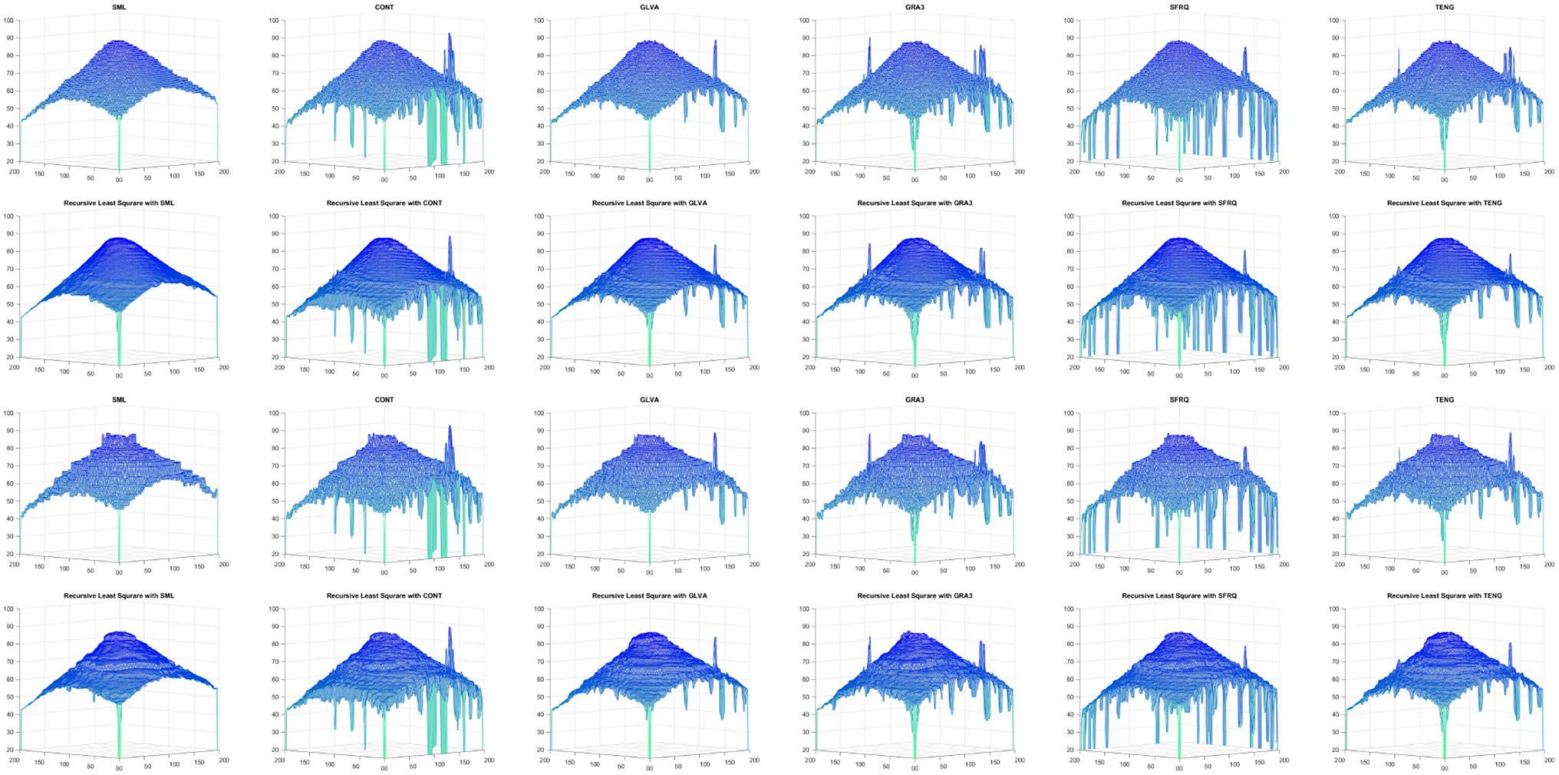
Shape reconstruction of real cone using FM with proposed RLS method with Jitter noise $\sigma ^2_\xi = (2ak+b)^2 \sigma ^2_{\zeta } + 2a ^2 \sigma ^4_{\zeta }$. (left to right), SML, CONT, GLVA, GRA3,SFRQ, TENG. (top row) with FM only with $\sigma _\zeta ^2 =0$, (second row) using FM with RLS method $\sigma _\zeta ^2 =0$, (third row) with FM only with $\sigma _\zeta ^2 =1.5$, (bottom row) using FM with RLS method $\sigma _\zeta ^2 =1.5$.

**Figure 12:**
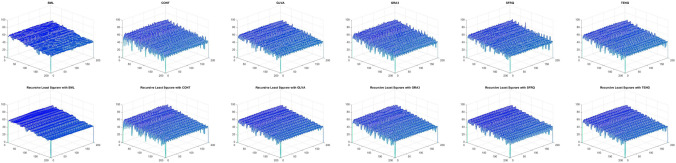
Shape reconstruction of Plane using FM with proposed RLS method. (left to right), SML, CONT, GLVA, GRA3,SFRQ, TENG. (top row) with FM only and (bottom row) using FM with RLS method.

**Figure 13:**
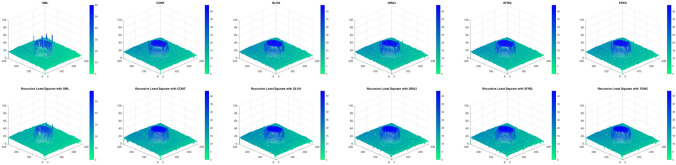
Shape reconstruction of LCD-TFT filter using FM with proposed RLS method. (left to right), SML, CONT, GLVA, GRA3,SFRQ, TENG. (top row) with FM only and (bottom row) using FM with RLS method.

**Figure 14:**
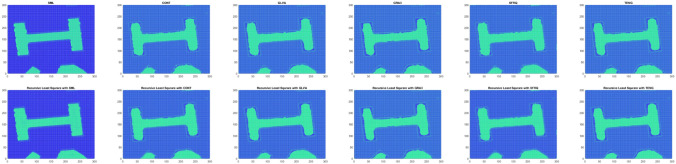
Shape reconstruction of Image-I using FM with proposed RLS method. (left to right), SML, CONT, GLVA, GRA3,SFRQ, TENG. (top row) with FM only and (bottom row) using FM with RLS method.

**Figure 15:**
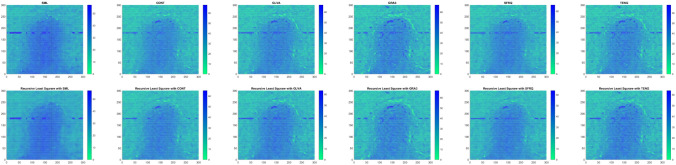
Shape reconstruction of Coin using FM with proposed RLS method. (left to right), SML, CONT, GLVA, GRA3,SFRQ, TENG. (top row) with FM only and (bottom row) using FM with RLS method.

Table 10 shows the results of Real Cone shape reconstruction for different levels of noise. These results show the similar trends as the simulated cone. When only FMs are used, the shape reconstruction deteriorates significantly with an increase in noise, whereas when these FMs are combined with the proposed RLS technique, the noise can be filtered out resulting in better shape reconstruction. Figure 11 represents the reconstructed shape of Real Cone using different FMs only and FMs with the proposed RLS technique. When the noise increases, the surface roughness is clearly visible in the figure when only FMs are used, whereas when RLS is combined with these FMs the surface roughness is significantly reduced. The shape reconstruction results of Real Plane, LCD-TFT filter, Image-I, and Coin, using only FMs and FMs with the proposed RLS filter are demonstrated in Figs. 12, 13, 14 and 15. It can be seen from the figures that reconstructed shapes of Real Cone and Real Plane are smoother. When using FM only, the roughness in shape was because of jittering, which is smoothed in the reconstructed shape by the application of the proposed method. The cylindrical shape of the filter is preserved in LCD-TFT filter, and the surrounding surface is smoothed by the filtering process. Jitter in the sequence of the data-set of Image-I is quite low, thus not much difference was observed visually. However, near the vertical axis of 175 value, a depth abnormality can be observed in shape reconstruction when using all the FMs, and also with the proposed scheme in the case of Coin sequence.

**Figure 16:**
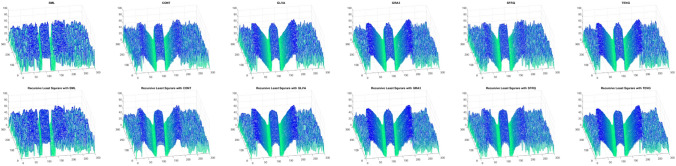
Shape reconstruction of Groove using FM with proposed RLS method. (left to right), SML, CONT, GLVA, GRA3,SFRQ, TENG. (top row) with FM only and (bottom row) using FM with RLS method.

The sides and center of groove are over-exposed, in the Groove image sequence. This causes texture degradation, which is critical in SFF systems. The change in focus levels is exhibited only by the slopes in the middle. Figure 16 shows the shape reconstruction results of the Groove using different FMs and FMs with the proposed filter application.

## Conclusion

Shape from focus is one of the passive techniques used to recover 3D shapes of the objects from their respective 2D images, by using the focus information present in the scene. SFF requires a huge number of images obtained at different focus levels, and the sampling step size for this image acquisition is taken as a constant. However, this inter-frame distance is susceptible to errors due to mechanical inaccuracies, such as flaws in gear assembly of the translational stage or lens assembly of the focusing device. These errors are termed as Jitter noise. Jitter noise is not visible in images, because each pixel in an image is subjected to the same error in focus, and thus traditional techniques of image denoising do not work in this case.

In this paper, Jitter noise is modeled using the Quadratic function for focus curves, and a recursive least squares filter is designed to remove this noise from SFF systems. In RLS, a single new data point is analyzed in each algorithm iteration to improve the estimation of the model parameters. Since it converges faster, an RLS filter is designed for shape from focus systems to remove the Jitter noise from the focus curves. To check the robustness of the results the proposed manuscript employs the RMSE and correlation and proposes a new metric referred to as the Depth Distortion metric, which indicates the number of pixels in percentage contributing to the RMSE. Seven objects are used for experiments herein: *one* simulated and *six* real. *Ten* noise levels are tested on the simulated object and *four* levels on the real objects. The experimental results validate the effectiveness of the proposed scheme.

**Table 10:** RMSE Correlation (Cor.) and Depth Distortion (D.D. in %) Metrics for Real Cone shape reconstruction using FMs with different levels of variance of Jitter noise $\sigma ^2_\xi = (2ak+b)^2 \sigma ^2_{\zeta } + 2a ^2 \sigma ^4_{\zeta }$ with ($\sigma _\zeta ^2 : 0, 0.1, 0.25, 0.5, 0.75, 1.0$).

FM used	$\sigma _\zeta ^2$	Corr.		RMSE		D.D.	
		With FM only	With RLS & FM	With FM only	With RLS & FM	With FM only	With RLS & FM
TENG	0	0.924	0.926	3.843	3.774	0.735	0.435
	0.1	0.924	0.926	3.826	3.765	0.690	0.430
	0.25	0.924	0.926	3.836	3.775	0.662	0.438
	0.5	0.923	0.925	3.853	3.798	0.733	0.423
	0.75	0.918	0.923	3.935	3.856	1.058	0.470
SML	0	0.927	0.927	3.711	3.693	0.975	0.120
	0.1	0.926	0.927	3.717	3.690	0.950	0.225
	0.25	0.928	0.927	3.725	3.703	1.418	0.267
	0.5	0.927	0.928	3.735	3.693	1.375	0.238
	0.75	0.863	0.885	5.220	4.725	0.143	0.118
GLVA	0	0.926	0.927	3.760	3.725	0.418	0.272
	0.1	0.926	0.928	3.756	3.714	0.418	0.285
	0.25	0.927	0.927	3.771	3.746	0.435	0.275
	0.5	0.925	0.926	3.775	3.760	0.543	0.267
	0.75	0.891	0.915	4.601	4.047	0.265	0.243
SFRQ	0	0.904	0.903	4.483	4.527	0.660	0.562
	0.1	0.905	0.905	4.443	4.459	0.653	0.560
	0.25	0.907	0.906	4.411	4.447	0.640	0.580
	0.5	0.901	0.901	4.530	4.560	0.600	0.528
	0.75	0.860	0.891	5.435	4.828	0.625	0.585
CONT	0	0.923	0.927	3.866	3.799	0.980	0.692
	0.1	0.923	0.927	3.886	3.804	0.955	0.708
	0.25	0.923	0.926	3.908	3.832	0.980	0.692
	0.5	0.922	0.926	3.879	3.808	0.935	0.685
	0.75	0.905	0.927	4.290	3.829	0.892	0.590
GRA3	0	0.919	0.924	3.976	3.838	1.015	0.665
	0.1	0.919	0.924	3.966	3.833	1.013	0.680
	0.25	0.920	0.924	3.988	3.843	1.135	0.730
	0.5	0.917	0.923	4.019	3.842	1.085	0.680
	0.75	0.911	0.922	4.104	3.924	1.053	0.672

## Data Availability

The datasets used and analyzed during the current study is available from the corresponding author on reasonable request.
